# Loop technique for anterior cruciate ligament reconstruction combined with anterolateral structure reinforcement: technical description and clinical results

**DOI:** 10.1186/s12893-024-02439-7

**Published:** 2024-06-14

**Authors:** Yang Xing, Aishan He, Yan Kang, Zibo Yang, Fangang Meng, Peihui Wu

**Affiliations:** Department of Sports Medicine, The First Affiliated Hospital of Sun Yat-sen University, No.58 Zhongshan Second Road, Guangzhou, 510080 China

**Keywords:** Anterior cruciate ligament, Anterolateral structure, Arthroscopy, Pivot shift

## Abstract

**Purpose:**

We describe a surgical technique for ACL reconstruction combined with anterolateral structure reinforcement and report early clinical follow-up results.

**Methods:**

The semitendinosus and gracilis tendons are braided into 5 strands and the ACL femoral tunnel and tibial tunnel are created. The graft is passed through the tunnel with the use of a traction suture and the tibial end is fixed with absorbable interference screws at 30° of knee flexion. The ACL graft traction suture is used as an anterolateral reconstruction structure to pass through the proximal exit of the ACL femoral tunnel and then through the depth of the iliotibial bundle to the anterior to Gerdy’s tubercle, a bony tunnel is created from the anterior to Gerdy’s tubercle to the goose foot, and the traction suture is passed through this bony tunnel to form a Loop structure at 20° of knee flexion. Between March 2021 and May 2022 IKDC score, Lysholm score, and Tegner score were performed preoperatively and 6–12 months postoperatively in 24 consecutive patients who met the indications for this procedure and underwent surgery. The patient’s maximum flexion angle, the circumference of the thigh, and the stress X-ray between the operated and healthy knee were measured.

**Results:**

Patients showed significant improvement in IKDC score, Lysholm score and Tegner score at a mean follow-up of 7 months postoperatively compared to preoperatively. No significant increase in anterior tibial displacement was found between the patient’s operated side and the healthy side.

**Conclusion:**

The Loop technique ACLR combined with ALSA can be used in patients with an ACL tear combined with a high degree of positive pivot shift. The patient’s subjective perception was significantly improved from the preoperative period and knee stability was restored.

**Level of evidence:**

IV, therapeutic study.

**Supplementary Information:**

The online version contains supplementary material available at 10.1186/s12893-024-02439-7.

## Introduction

The anterior cruciate ligament (ACL) is the most commonly injured ligament of the knee [1], impacting over 200,000 people in the US each year [2]. ACL reconstruction (ACLR) is the most effective treatment for ACL tears, however, the failure rate after 5 years is 53% and residual rotational instability after ACLR can lead to deterioration of knee function and patient dissatisfaction [3, 4].

Studies have shown that one of the reasons for failure after ACLR is the ignoring of damage to the anterolateral structures (ALS) [5, 6], ALS consists of the iliotibial bundle, the anterolateral joint capsule and the anterolateral ligament(ALL), which help the ACL maintain rotational stability of the knee [7, 8, 9], ACLR combined with ALSR shows inspiring clinical results [10, 11].

However, the ALSR technique described previously in the publication requires additional autologous tendons or the use of artificial ligaments, which increases the trauma to the patient and the patient’s hospitalization costs. We developed a new and simple ALS reinforcement (ALSR) technique using a graft traction suture as a reinforcing structure without additional tendon retrieval to effectively control the rotational instability that may exist after ACLR. We hypothesize that this surgical technique will provide satisfactory clinical results.

## Methods

Patients who underwent ACLR combined with ALSA at our institution between March 2021 and May 2022 were included in this study, and patients’ data were collected retrospectively. This study was performed in line with the principles of the Declaration of Helsinki. Approval was granted by the Ethics Committee of our institution. All operations were performed by one surgeon at the same institution. Indications for the surgery were: (1) Chronic ACL tear for more than 1 year; (2) Positive grade-II, III pivot test; (3) High demands for sports. All clinical scores, physical examinations, and imaging examinations were obtained with patients’ informed consent.

## Surgical technique

The patient is placed in the supine position and an appropriate tourniquet is applied to the plaster pad. The lateral column is positioned at the level of the tourniquet to prevent outward rotation of the hip.

### Arthroscopic exploration

Standard anteromedial and anterolateral arthroscopic portals are established. Standard arthroscopy is performed to assess associated meniscal and cartilage damage and treat if necessary. ACL tension and integrity are then probed.

### Graft preparation

A longitudinal incision is made 2 cm below the goose foot within the tibial tuberosity, the semitendinosus and gracilis are identified and separated, cut vertically at the tibial stop, and the tendons are obtained separately with a tendon extractor. The length of the tendon was checked and the tendon was sutured at the end with a non-absorbable No.0 suture. A 5-strand graft is made and the diameter of the graft is measured. The femoral end is fixed with a button plate and traction suture through it (Arthrex, Naples, USA), and the tibial end is braided with non-absorbable No. 2 sutures.

### Creating a femoral tunnel and femoral skin incision

**Fig. 1:**
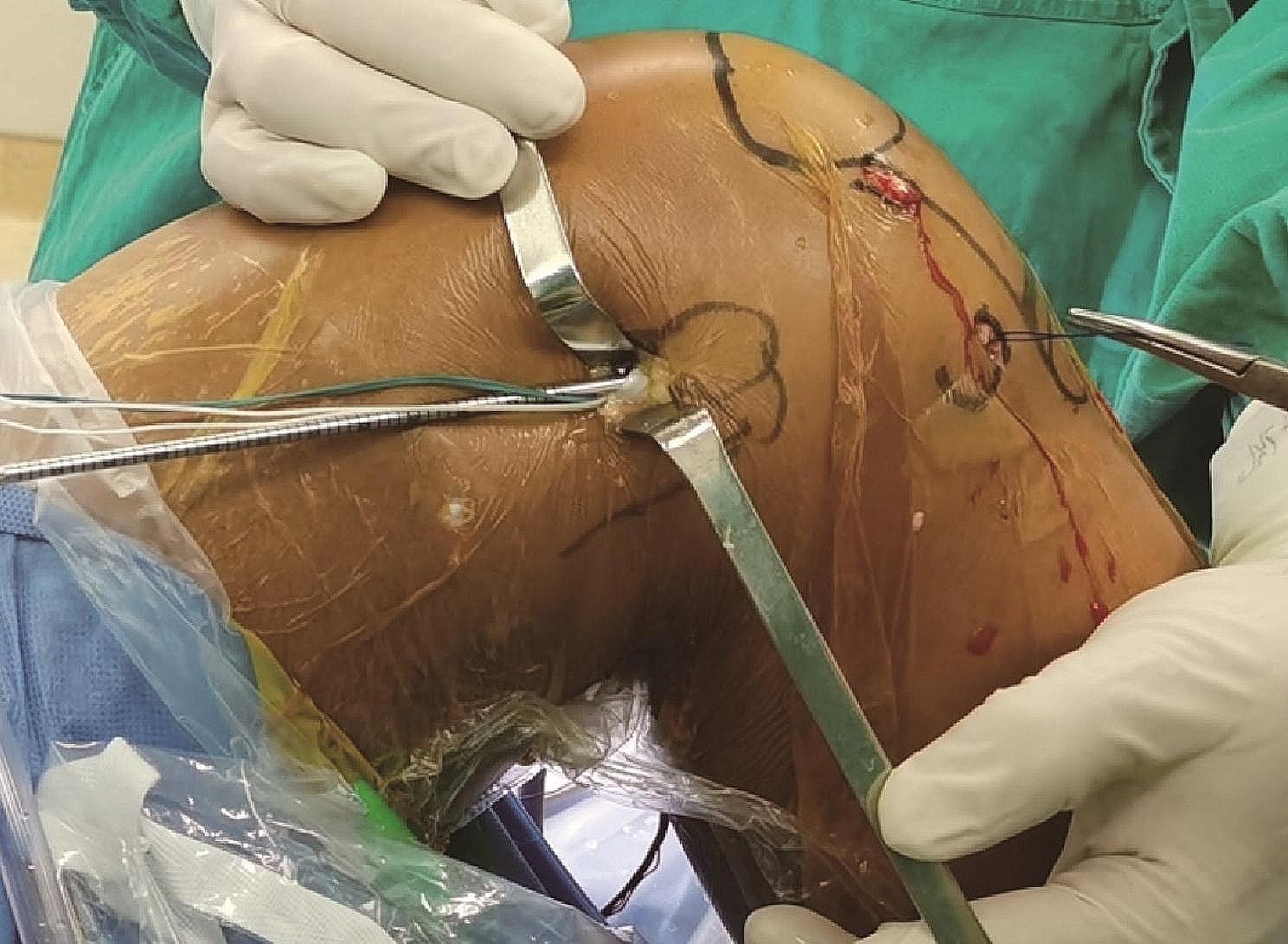
The femoral tunnel is created using an inside-out method so that the proximal exit of the femoral tunnel is located approximately 1 cm proximal and posterior to the lateral epicondyle of the femur

The femoral tunnel is created in the original ACL footprint area through the anteromedial portal, using an inside-out approach according to the diameter of the graft, making the proximal exit of the femoral tunnel located at the proximal posterior side of the lateral epicondyle of the femur. A skin incision of approximately 1 cm is made at the lateral epicondyle of the femur to reach the deep layer of the iliotibial bundle. Insert the oval forceps between the deep layer of the iliotibial bundle and the cortical bone, adjust the position of the oval forceps so that the K-wire is passed through it. This K-wire is used as a guide so that the Ethibond No. 2 suture is passed through the femoral tunnel and the oval forceps (Fig. 1).

### Creating a tibial tunnel for ACL

Using a tibial locator, the distal exit of the tibial tunnel is positioned at the point where the semitendinosus and thin femoral muscle incisions are taken and the intra-articular exit is positioned at the middle of the original ACL tibial attachment point where the ACL stump is present, creating the tibial tunnel in an outside-in method. The tibial portal is enlarged according to the diameter of the graft, and the Ethibond No. 2 suture is passed through the tibial tunnel into the joint using a circlip clamp and threaded out of the tibial tunnel.

### Passage and fixation of the graft and reinforcement structure

**Fig. 2:**
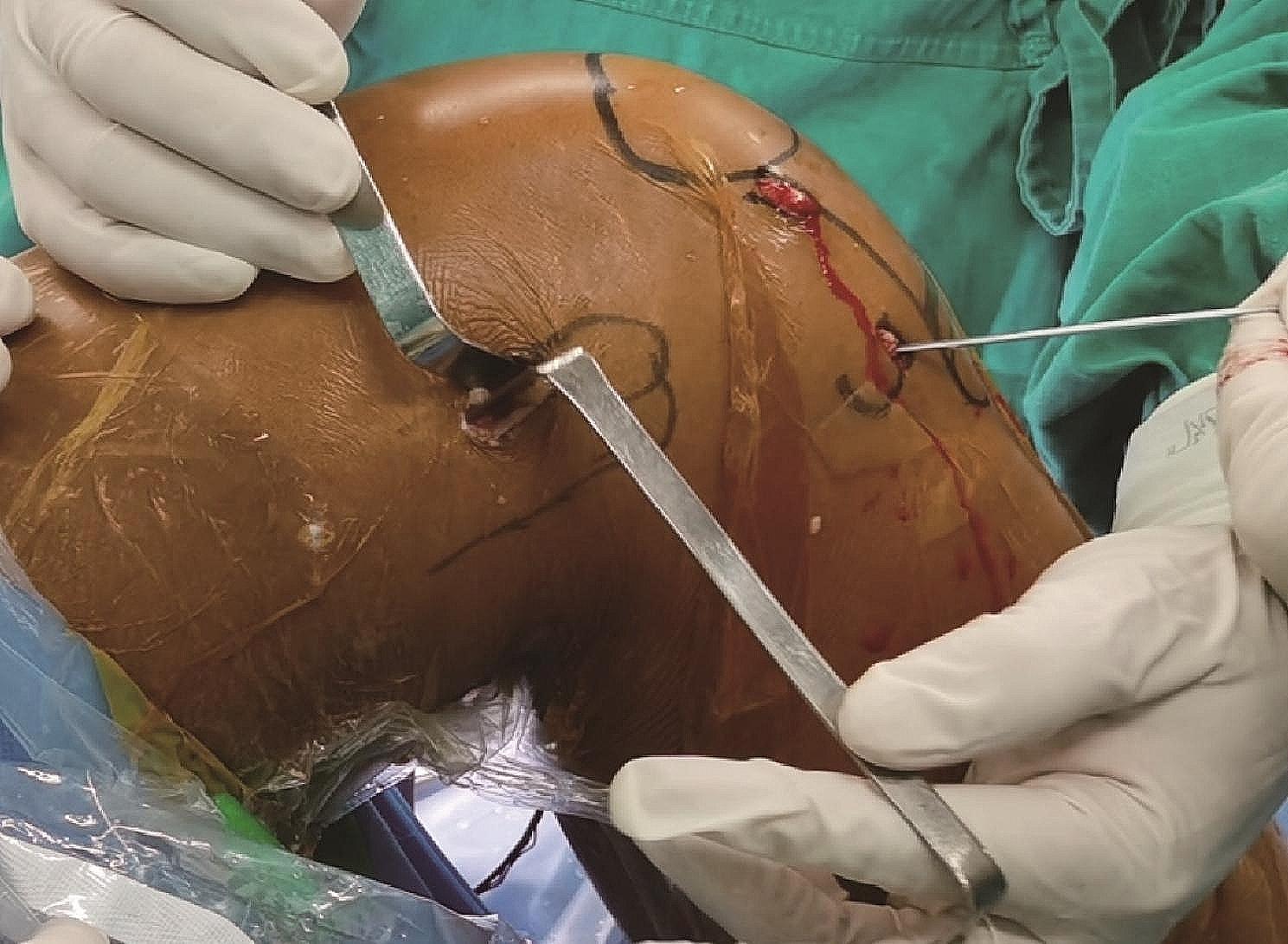
A channel is created in the depth of the iliotibial bundle from the exit of the femoral tunnel to the anterior of Gerdy’s tubercle, so that the anterolateral reinforcement structures pass through it

**Fig. 3:**
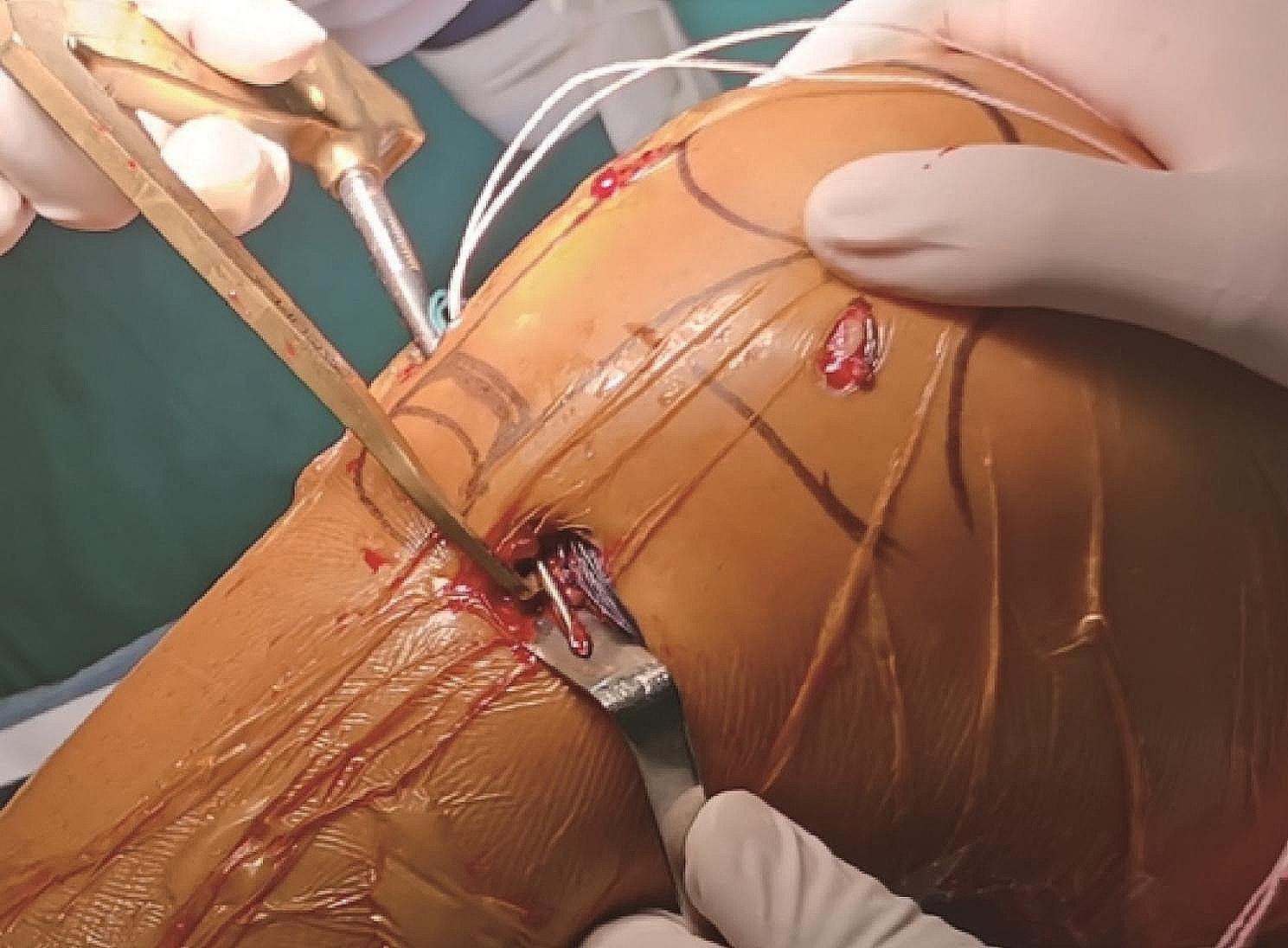
A bony channel is created from the anterior of Gerdy’s tubercle to the goosefoot so that the traction suture passes through it

**Fig. 4:**
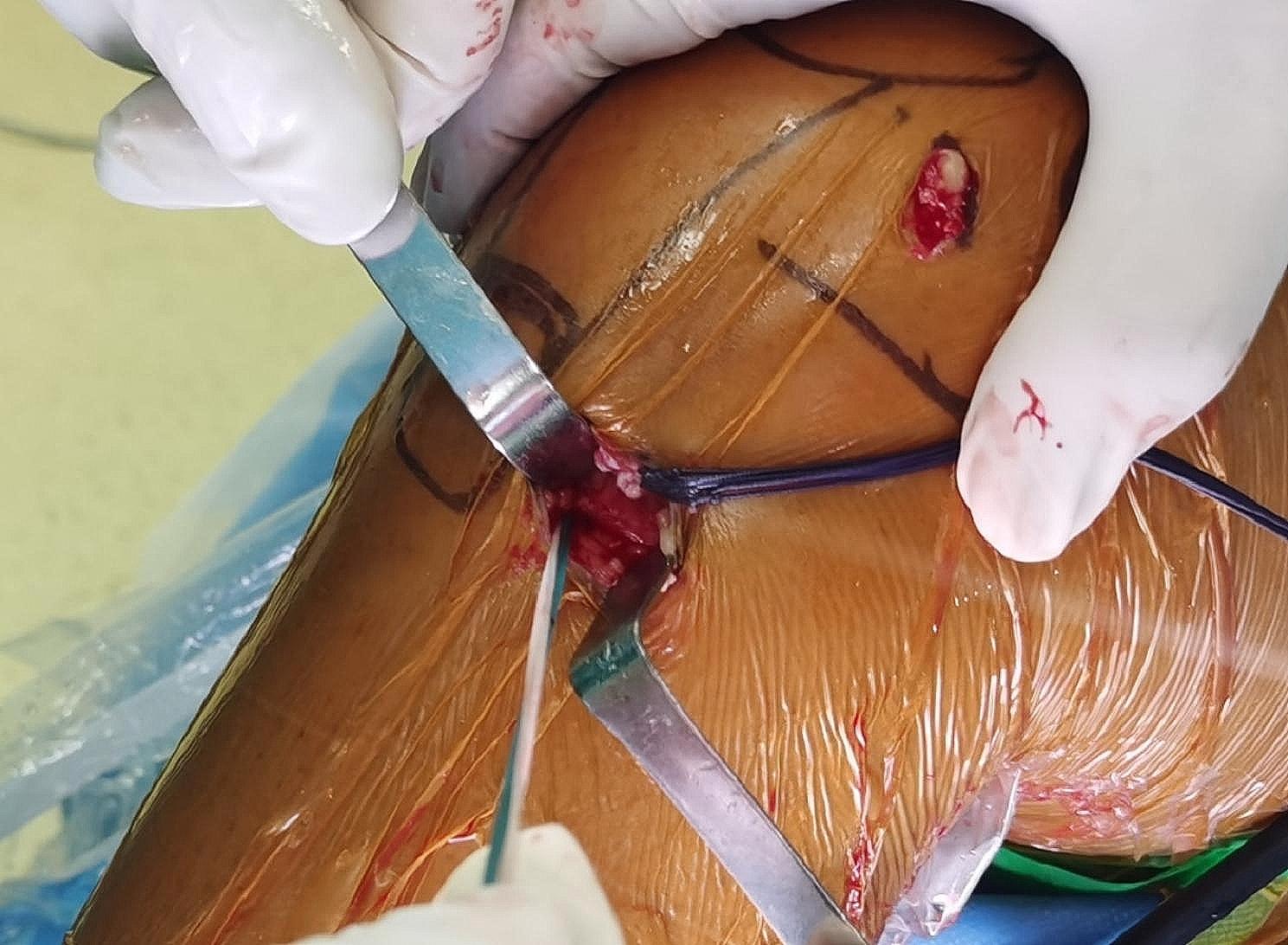
The traction suture is sutured to the stump of the distal tibia of the graft to fix it and form a Loop structure

**Fig. 5:**
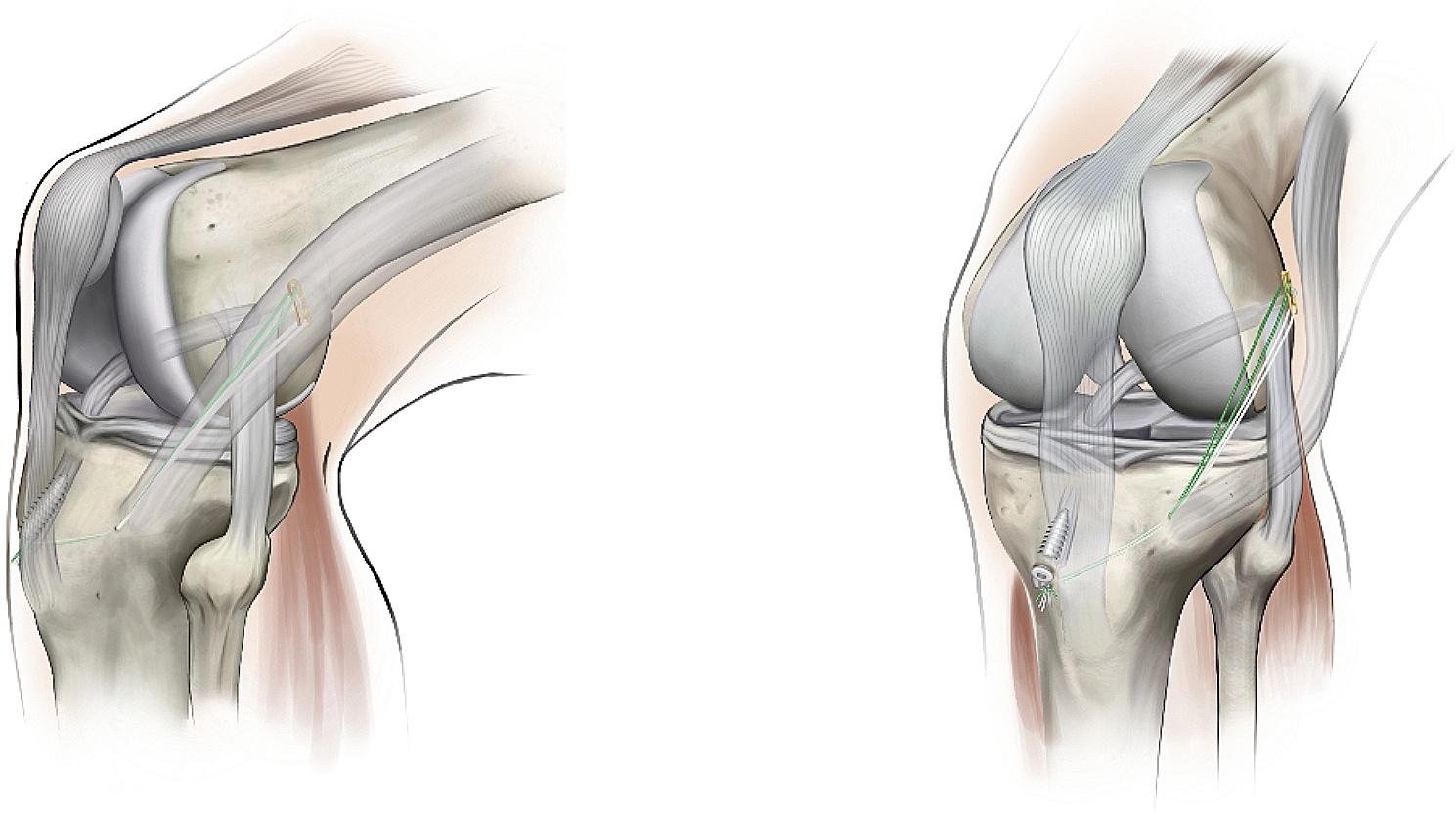
Diagram of the Loop technique: the ACL graft traction suture exits the proximal femoral tunnel through the depth of the iliotibial bundle to the anterior of Gerdy’s tubercle to form the ALS reinforcement structure

The Ethibond No. 2 suture, which passes through the femoral and tibial tunnels, is used as a guide with the help of which the graft is passed sequentially through the tibial tunnel, intra-articular, and femoral tunnel, so that the button plate is fixed at the proximal exit of the femoral tunnel. The knee is flexed at 30° to pull the tibial end of the graft taut and the tibial end of the graft is fixed using absorbable interference screws. At this point the traction suture is passed through the proximal exit of the femoral tunnel and out through the skin through the oval clamp and the traction suture is pulled out through the femoral skin incision using the oval clamp. A 1 cm longitudinal incision is made anterior to Gerdy’s tubercle reaching the deep level of the iliotibial bundle. A wire clamp is used to pass through the deep level of the iliotibial bundle to the proximal exit of the femoral tunnel, and the traction suture at the proximal end of the femoral tunnel is pulled anterior to Gerdy’s tubercle (Fig. 2). A bony tunnel is created with a tibial tunnel locator from anterior to Gerdy’s tubercle to just below the ACL tibial tunnel, and a guide pin is used to pull the traction suture from anterior to Gerdy’s tubercle to the distal exit of the tibial tunnel (Fig. 3). The traction suture is sutured together with the distal remnants of the graft at the tibia at 20° of knee flexion so as to form a loop structure (Figs. 4 and 5).

### Postoperative rehabilitation

Patients returned to the outpatient department at 6 weeks, 3 months, and 6 months after the operation for follow-up visits and appropriate rehabilitation instructions. Progressive range of motion exercises were performed immediately after the operation, with flexion of the knee to a maximum of 90° and partial weight bearing with a crutch permitted for the first 6 weeks. The knee flexion angle is not limited after 6 weeks. Patients are allowed to perform sports such as swimming and cycling at 3 months after the operation, and specific sports training is allowed at 6 months.

### Assessments

The IKDC score, Lysholm score and Tegner score were used to assess the clinical outcome of the patients in the early postoperative period (6–12 months). The difference in the maximum knee flexion angle between the healthy side and the operated side and the ratio of the operated side to the healthy side of the 15 cm circumference of the thigh above the patella were also measured at the last follow-up.

**Fig. 6:**
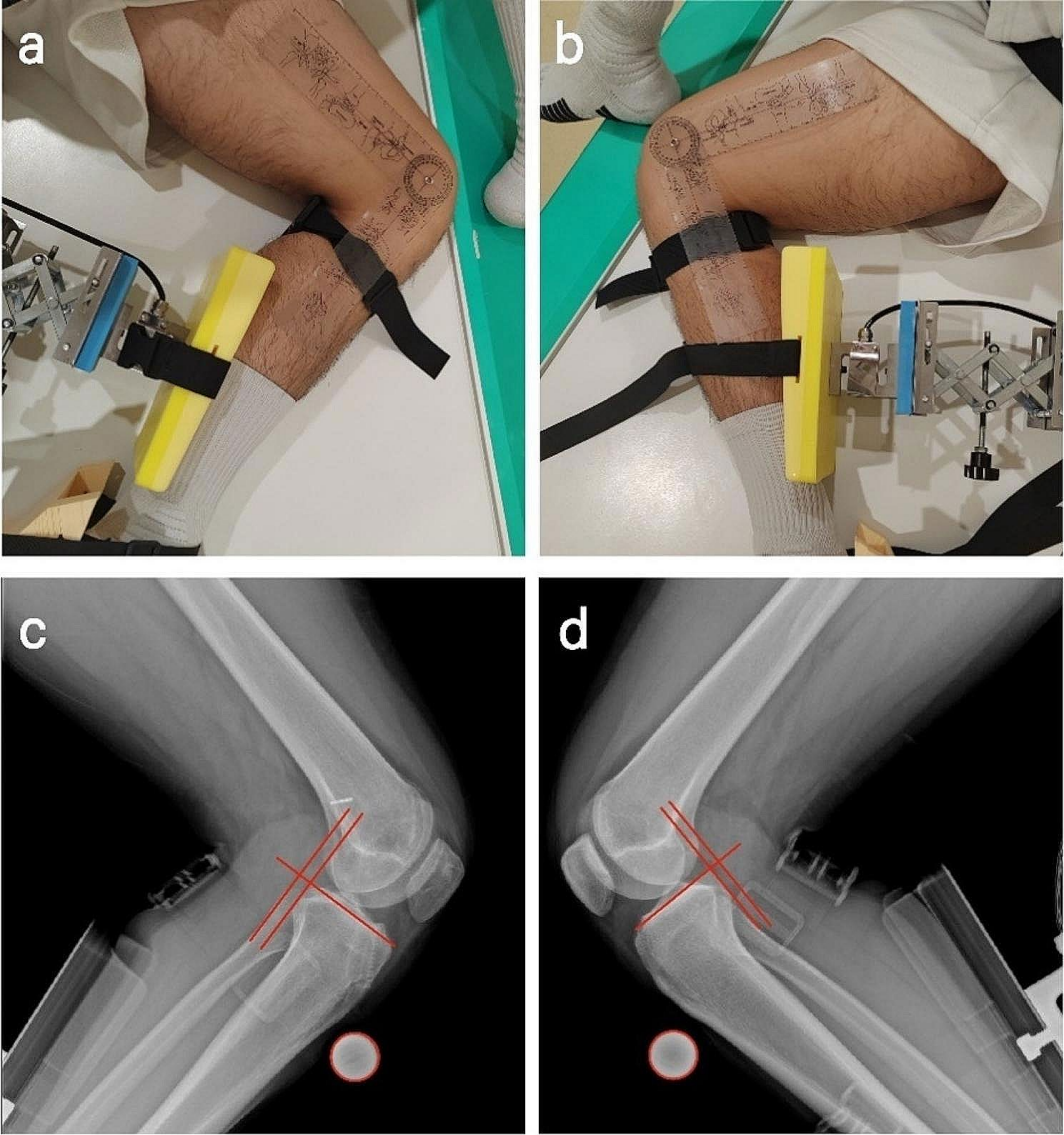
Stress radiographs were taken at 90° flexion: (**a**)left and (**b**)right knee. Perpendicular lines from the reference line (parallel to the medial tibial plateau joint line) were drawn tangentially to the posterior contour of the medial femoral condyle and the posterior contour of the medial tibial plateau. The distance between the medial femoral condyle and the medial tibial plateau was measured to calculate the side-to-side differences: (**c**) and (**d**)

Stress radiographs were taken by a licensed radiologic technologist with the use of a stress position device at the postoperative follow-up. The patient was placed in a lateral position, the hip and ankle joints of the imaged leg were fastened with straps, a 25 mm diameter metallic positioning ball was placed in front of the tibial tuberosity, the knee is flexed at 90°, a force of 134 N was applied with the assistance of a pressure transducer at the posterior side of the tibia, and bilateral knee radiographs were performed [12, 13, 14] (Fig. 6a, b).

All radiological measurements were processed and measured using the Siemens software package (Syngo, Siemens Medical Solutions, Erlangen, Germany). Calculate the side-to-side difference of anterior tibial translation (ATT) between the operated and non-operated sides. The method of measurement was as described previously [14], a straight line was drawn along the tibial plateau and a vertical line was drawn tangent to the posterior edge of the medial tibial plateau, the distance from the posterior edge of the medial femoral condyle to this line was measured, and the value of this was taken as the ATT of this side, and the difference between the ATT on the operative side and the non-operative side as the SSD (Fig. 6c, d).

### Statistical analysis

Data analysis was performed using SPSS 26.0 (IBM, Armonk, New York, USA). Descriptive statistics are presented as mean ± standard deviation (SD) for all continuous variables. The quantitative data were first subjected to the Shapiro-Wilk test to determine whether the data conformed to the normal distribution, and when comparing the differences between the two groups, the Student t test was used if the data conformed to a normal distribution, and the nonparametric Mann-Whitney U test was used if the data did not conform to a normal distribution. Paired t test was used for preoperative and last follow-up subjective scores and knee flexion range and thigh circumference. The statistical significance level was set at 0.05.

## Results

A total of 24 patients, including 19 males and 5 females, were included in this study. The mean follow-up time was 7.0 ± 1.1 months, the mean age of the patients was 25.6 ± 7.3 years, and the mean BMI was 24.1 ± 3.4 kg/m2. 21 patients with associated medial meniscus injury; 17 patients with associated lateral meniscus injury. At a mean of 7 months postoperatively, patients’ mean IKDC score increased from 53.9 ± 5.6 to 75.8 ± 2.4 (*p* < 0.05), mean Lysholm score increased from 60.5 ± 5.1 to 90.3 ± 1.7 (*p* < 0.05) and mean Tegner score increased from preoperative 3.1 ± 0.4 to 4.5 ± 0.2 (*p* < 0.05). The thigh circumference ratio of the operated side to the healthy side was 95.8 ± 0.5%, the maximum bending angle on the operated side was 4.6 ± 0.9° less than on the healthy side, and the maximum bending angle of the knee on the operated side was over 130° in all patients. The stress x-ray examination with the knee flexed at 90° showed a mean SSD of -0.2 ± 1.9 mm. No patient was found to have anterior tibial displacement of more than 3 mm. No complications were found in all patients in the early postoperative period (Table 1).

## Discussion

The main result of this study is the proposal of a new ACLR combined with ALSR technique and the confirmation of the effectiveness of this surgical approach by subjective pre- and early post-operative scoring of patients. The patients’ subjective scores were significantly better than pre-operative, and no significant difference in knee stability was seen compared to the healthy side, with all patients having an SSD of less than 3 mm.

The aim of ACLR surgery is to restore stability of the knee especially rotational stability, however some studies have reported residual rotational instability of 11–30% after ACLR [15, 16].

Unlike the previously described ALSR technique [17, 18, 19–21], the Loop technique uses a graft traction suture to connect the femoral tunnel exit to the front of Gerdy’s tubercle. It is therefore not limited by one’s own ligament length, does not require additional retrieval of the peroneus longus tendon, avoids possible injury and is simple and easy to perform. In addition, because of the reliable strength of the traction suture, it facilitates early functional rehabilitation and the return of the patient’s pre-injury sports.

Many cadaveric anatomical studies of the anterolateral ligament have been performed, showing a preference for locating the ALL femoral stop at the posterior proximal aspect of the lateral femoral epicondyle, despite the wide variation in its termination point [22, 23, 24, 25], and biomechanical tests suggest that the ALSR femoral position at the posterior proximal aspect of the lateral epicondyle restores better rotational stability of the knee joint, with better isometric properties and less variation in tension [26, 9, 27]. Previous biomechanical studies have shown that when the femoral stop of the ALSR graft is positioned at the posterior proximal end of the lateral epicondyle of the femur, no statistical difference in anterior tibial displacement was seen between different femoral stops simulating the anterior drawer test [9]. This surgical approach therefore positions the proximal exit of the ACL femoral tunnel posterior to the proximal end of the lateral epicondyle of the femur, so that the exit of the ACL femoral tunnel coincides with the point of attachment of the reinforcing structure to the femur, avoiding additional fixation of the ALS at the femoral end.

In conclusion, we would like to propose a simpler and easier to master extra-articular ALS-enhanced Loop technique to replace the traditional ACLR technique, in order to compensate for the shortcomings of the ACLR technique for controlling rotation instability.

**Table 1:** Patient characteristics

	Patient
Age, y	25.6 ± 7.3
BMI, kg/m2	23.9 ± 3.3
Sex, male/female	19/5
Follow up time, m	7.0 ± 1.3
Associated injuries	
Medial meniscus	21
Lateral meniscus	17
Side-to-side difference, mm	-0.2 ± 1.9

### Electronic supplementary material

Below is the link to the electronic supplementary material.

Supplementary Material 1

## Data Availability

All data generated or analyzed during this study are included in this published article [and its supplementary information files].

## References

[1] Granan LP, Forssblad M, Lind M, Engebretsen L (2009). The scandinavian ACL registries 2004–2007: baseline epidemiology. Acta Orthop.

[2] Kaeding CC, Leger-St-Jean B, Magnussen RA (2017). Epidemiology and diagnosis of Anterior Cruciate Ligament injuries. Clin Sports Med.

[3] Ayeni OR, Chahal M, Tran MN, Sprague S (2012). Pivot shift as an outcome measure for ACL reconstruction: a systematic review. Knee Surg Sports Traumatol Arthrosc.

[4] Feagin JA, Curl WW (1976). Isolated tear of the anterior cruciate ligament: 5-year follow-up study. Am J Sports Med.

[5] Kittl C, El-Daou H, Athwal KK, Gupte CM, Weiler A, Williams A, Amis AA (2016). The role of the Anterolateral structures and the ACL in Controlling Laxity of the Intact and ACL-Deficient knee. Am J Sports Med.

[6] Musahl V, Getgood A, Neyret P, Claes S, Burnham JM, Batailler C, Sonnery-Cottet B, Williams A, Amis A, Zaffagnini S, Karlsson J (2017). Contributions of the anterolateral complex and the anterolateral ligament to rotatory knee stability in the setting of ACL Injury: a roundtable discussion. Knee Surg Sports Traumatol Arthrosc.

[7] Getgood A, Brown C, Lording T, Amis A, Claes S, Geeslin A, Musahl V, Group ALCC (2019). The anterolateral complex of the knee: results from the International ALC Consensus Group Meeting. Knee Surg Sports Traumatol Arthrosc.

[8] Herbst E, Albers M, Burnham JM, Shaikh HS, Naendrup JH, Fu FH, Musahl V (2017). The anterolateral complex of the knee: a pictorial essay. Knee Surg Sports Traumatol Arthrosc.

[9] Katakura M, Koga H, Nakamura T, Araki D, Nagai K, Nishida K, Kuroda R, Muneta T (2019). Biomechanical effects of additional Anterolateral structure Reconstruction with different femoral attachment sites on Anterior Cruciate Ligament Reconstruction. Am J Sports Med.

[10] Hurley ET, Fried JW, Kingery MT, Strauss EJ, Alaia MJ (2021). Antero-lateral ligament reconstruction improves knee stability alongside anterior cruciate ligament reconstruction. Knee Surg Sports Traumatol Arthrosc.

[11] Yoon KH, Hwang IU, Kim EJ, Kwon YB, Kim SG (2021). Anterolateral Ligament Reconstruction Improves Anteroposterior Stability as Well as Rotational Stability in Revision Anterior Cruciate Ligament Reconstruction with High-Grade Pivot Shift. J Knee Surg.

[12] Delaloye JR, Hartog C, Blatter S, Schlappi M, Muller D, Denzler D, Murar J, Koch PP (2020). Anterolateral Ligament Reconstruction and Modified Lemaire lateral extra-articular tenodesis similarly improve knee Stability after Anterior Cruciate Ligament Reconstruction: a Biomechanical Study. Arthroscopy.

[13] James EW, Williams BT, LaPrade RF (2014). Stress radiography for the diagnosis of knee ligament injuries: a systematic review. Clin Orthop Relat Res.

[14] Lee HJ, Park YB, Kim SH (2019). Diagnostic Value of Stress Radiography and arthrometer measurement for anterior instability in anterior cruciate ligament injured knees at different knee flexion position. Arthroscopy.

[15] Park YB, Lee HJ, Ro DH, Lee GY, Kim S, Kim SH (2019). Anterolateral ligament injury has a synergic impact on the anterolateral rotatory laxity in acute anterior cruciate ligament-injured knees. Knee Surg Sports Traumatol Arthrosc.

[16] Tashman S, Collon D, Anderson K, Kolowich P, Anderst W (2004). Abnormal rotational knee motion during running after anterior cruciate ligament reconstruction. Am J Sports Med.

[17] Helito CP, Bonadio MB, Gobbi RG, E.A.R.F. da Mota JR, Pecora GL, Camanho, Demange MK (2015). Combined intra- and Extra-articular Reconstruction of the anterior cruciate ligament: the Reconstruction of the knee anterolateral ligament. Arthrosc Tech.

[18] Kernkamp WA, van de Velde SK, Bakker EW, van Arkel ER (2015). Anterolateral Extra-articular Soft tissue Reconstruction in Anterolateral Rotatory instability of the knee. Arthrosc Tech.

[19] Smith JO, Yasen SK, Lord B, Wilson AJ (2015). Combined anterolateral ligament and anatomic anterior cruciate ligament reconstruction of the knee. Knee Surg Sports Traumatol Arthrosc.

[20] Sonnery-Cottet B, Saithna A, Cavalier M, Kajetanek C, Temponi EF, Daggett M, Helito CP, Thaunat M (2017). Anterolateral Ligament Reconstruction is Associated with significantly reduced ACL graft rupture rates at a Minimum follow-up of 2 years: a prospective comparative study of 502 patients from the SANTI Study Group. Am J Sports Med.

[21] Sonnery-Cottet B, Thaunat M, Freychet B, Pupim BH, Murphy CG, Claes S (2015). Outcome of a combined anterior cruciate ligament and Anterolateral Ligament Reconstruction technique with a Minimum 2-Year follow-up. Am J Sports Med.

[22] Ariel de Lima D, Helito CP, Lacerda de Lima L, de Castro Silva D, Costa ML, Cavalcante, Dias Leite JA (2019). Anatomy of the Anterolateral ligament of the knee. Syst Rev Arthrosc.

[23] Daggett M, Ockuly AC, Cullen M, Busch K, Lutz C, Imbert P, Sonnery-Cottet B (2016). Femoral origin of the Anterolateral ligament: an anatomic analysis. Arthroscopy.

[24] Farhan PHS, Sudhakaran R, Thilak J (2017). Solving the mystery of the Antero lateral ligament. J Clin Diagn Res.

[25] Helito CP, do Prado Torres JA, Bonadio MB, Aragao JA, de Oliveira LN, Natalino RJ, Pecora JR, Camanho GL, Demange MK (2017). Anterolateral ligament of the fetal knee: an anatomic and histological study. Am J Sports Med.

[26] Katakura M, Koga H, Nakamura K, Sekiya I, Muneta T (2017). Effects of different femoral tunnel positions on tension changes in anterolateral ligament reconstruction. Knee Surg Sports Traumatol Arthrosc.

[27] Kittl C, Halewood C, Stephen JM, Gupte CM, Weiler A, Williams A, Amis AA (2015). Length change patterns in the lateral extra-articular structures of the knee and related reconstructions. Am J Sports Med.

